# Photocatalytic Degradation of Organic Dye under UV-A Irradiation Using TiO_2_-Vetiver Multifunctional Nano Particles

**DOI:** 10.3390/ma10020122

**Published:** 2017-01-30

**Authors:** Le Thi Song Thao, Trinh Trung Tri Dang, Wilawan Khanitchaidecha, Duangdao Channei, Auppatham Nakaruk

**Affiliations:** 1 Department of Civil Engineering, Faculty of Engineering, Naresuan University, Phitsanulok 65000, Thailand; janetle.176@gmail.com (L.T.S.T.); tttdang247@gmail.com (T.T.T.D.); wilawank1@gmail.com (W.K.); 2 Centre of Excellence for Innovation and Technology for Water Treatment, Naresuan University, Phitsanulok 65000, Thailand; 3 Department of Chemistry, Faculty of Science, Naresuan University, Phitsanulok 65000, Thailand; duangdaoc@nu.ac.th; 4 Research Center for Academic Excellence in Petroleum, Petrochemicals and Advanced Materials, Naresuan University, Phitsanulok 65000, Thailand; 5 Department of Industrial Engineering, Faculty of Engineering, Naresuan University, Phitsanulok 65000, Thailand

**Keywords:** TiO_2_, vetiver grass, core–shell, photocatalysis, carbon reduction, nanostructure

## Abstract

The properties and photocatalytic performance of anatase nanoparticles of pure TiO_2_ and a core–shell structure of TiO_2_ on calcined vetiver grass leaves have been compared. Samples were fabricated by sol-gel and heating at 450 °C for 5 h. The comparison was based on data for X-ray diffraction (XRD), UV-Vis spectrophotometry, photoluminescence, transmission electron microscopy, specific surface area measurement, pore volume assessment, and methylene blue degradation testing. The results showed that the pure TiO_2_ consisted of agglomerated equiaxed nanoparticles of individual grain sizes in the range 10–20 nm. In contrast, the TiO_2_-vetiver composite exhibited a core–shell structure consisting of a carbonaceous core and TiO_2_ shell of thickness 10–15 nm. These features influenced the photocatalytic performance in such a way that the lower cross-sectional area, greater surface area, and higher pore volume of the TiO_2_ shell increased the number of active sites, reduced the charge carrier diffusion distance, and reduced the recombination rate, thereby improving the photocatalytic activity. This improvement derived from morphological characteristics rather than crystallographic, semiconducting, or optical properties. The improved performance of the TiO_2_-vetiver core–shell was unexpected since the X-ray diffraction data showed that the crystallinity of the TiO_2_ was lower than that of the pure TiO_2_. These outcomes are attributed to the reducing effect of the carbon on the TiO_2_ during heating, thereby facilitating the formation of oxygen vacancies, which enhance charge separation and hence photocatalysis by TiO_2_.

## 1. Introduction

Water is an indispensable part of life because it is essential for various purposes, including drinking, public hygiene, energy, agriculture, and industry. However, freshwater resources currently are diminishing due to the increasing growth of global population, over-exploitation, and water pollution [1]. In order to deal with water scarcity, wastewater treatment and reclamation are favourable approaches because they can prevent water pollution, protect public health, and provide reclaimed water source for agriculture and industry [2].

The textile industries annually produce huge amounts of wastewater that contains organic dyes, which significantly contribute to global water pollution [3]. Most synthetic dyes used in the textile industries consist of aromatic ring structures, which make them toxic, chemically resistant, and non-biodegradable in the natural environment [4,5]. Therefore, removal of organic dyes is an important target in wastewater treatment and reclamation.

The key criterion in wastewater treatment and reclamation is the quality of the treated water so that it can be determined as safe for use. At present, some common technologies used in wastewater treatment include sedimentation, flocculation and coagulation, filtration, adsorption, and biological processes. These traditional wastewater treatment technologies have had a long history in large-scale applications and effectiveness for the removal of various pollutants, such as organic compounds, nutrients, pathogens, and heavy metals [6]. However, a significant drawback of these technologies is the generation of secondary waste, which requires additional post-treatment.

In order to overcome secondary waste generation from wastewater treatment and satisfy the stringent standards for water treatment and use, technologies based on photocatalysis have attracted considerable interest. Among the semiconductor photocatalysts, TiO_2_ is used widely owing to its high photoactivity, photocorrosion resistance, thermal stability, non-toxicity, cost effectiveness, and potential applications under UV light (300–390 nm) [7,8]. When excited by a light source of energy equal to or greater than the band gap of the TiO_2_ photocatalyst (3.0–3.2 eV [9]), electron and hole pairs are generated. The generated holes then can transfer to the surface of TiO_2_ particles and react with water to form hydroxyl radicals (•OH) and superoxide radicals (•O^2−^) [10]. These radicals then act as or react with the environment to produce reactive oxygen species (ROS) in the form of hydroxyl (•OH) groups, hyperperoxyl (•HO_2_) groups, and hydrogen peroxide (H_2_O_2_). These ROS have the advantage of being able to mineralise a wide range of organic compounds in wastewater into simpler organic products [11] or to decompose them completely into carbon dioxide and weak mineral acids [12,13,14].

The effectiveness of pollutant removal can be increased through the applications of the (1) surface modification [15,16,17]; (2) band gap narrowing [18,19,20]; and (3) co-mechanisms of adsorption by adsorbent and photocatalysis [21]. Consequently, there are many studies that have reported the immobilisation of TiO_2_ particles on adsorbents such as zeolites, activated carbon, silica, and clays [22,23,24,25,26]. Furthermore, the immobilisation of TiO_2_ on an adsorbent can help to reduce the wash-out of TiO_2_ into the treatment system [27].

Zeolites and activated carbon are two of the most commonly used adsorbents in wastewater treatment. Both can be synthesised from agricultural wastes, such as coconut shell, straw, rice husk, bamboo, sedge weed, pokeweed, and sugarcane bagasse; these approaches are attractive from the perspectives of cost effectiveness and reduction of waste generation [28,29]. Amongst agricultural wastes, vetiver grass (*Vetiveria zizanioides* (L.) Nash) is a potential precursor, as it is most frequently used for erosion control and oil extraction in many tropical and subtropical countries [30]. The fragrant and volatile oil extracted from the roots of vetiver grass is used commercially in a range of industries, including perfumery, cosmetics, soap, and pharmaceuticals [31]. In environmental applications, vetiver grass has shown an ability to accumulate persistent organic pollutants (POPs), polycyclic aromatic hydrocarbons (PAHs), phenol, and a variety of heavy metals from contaminated soil and water [31,32,33]. However, the principal uses of vetiver grass for erosion control and extracted oil production result in the production of a large amount of agricultural waste in the form of its unused leaves [34].

In the present work, vetiver leaves were used as a precursor to synthesise a multifunctional material combining the adsorption ability of the vetiver leaves and the photocatalytic activity of TiO_2_. The synthesised material was characterised for a range of physicochemical properties and its applicability to wastewater treatment assessed by degradation of the organic dye methylene blue (MB).2. Methodology.

### 2.1. Preparation of Vetiver Adsorbent

Vetiver grass was collected from the field in Phitsanulok Province, Thailand. Only the leaves of the vetiver grass were used to synthesise the adsorbent while other parts were removed. After cleaning with water to remove dirt, the vetiver leaves were dried in a muffle furnace at 105 °C for 12 h in order to remove moisture. The dried vetiver leaves were crushed with a porcelain mortar and pestle and sieved to a particle size in the range 0.1–0.6 mm, after which they were pyrolysed in the same muffle furnace at 600 °C for 3 h, followed by natural cooling. After pyrolysis, the residue was stored in an airtight container. The main inorganic components of the solid residue of vetiver leaves were determined previously to be potassium (45 wt%) and silicon (27 wt%) [35]. The physical and chemical properties of dried vetiver and adsorption mechanism were reported in the previous work [35].

### 2.2. Synthesis of Pure TiO_2_ and TiO_2_-Vetiver Core–Shell

The pure TiO_2_ and TiO_2_-vetiver core–shell was synthesised using a sol-gel method reported elsewhere [36]. For the latter, 1 g of calcined vetiver adsorbent was added to a mixture of 12.5 mL of titanium isopropoxide (TTIP), 80 mL of 2-propanol, and 3 mL of deionised (DI) water, after which blending was achieved by magnetic stirring for 4 h. The resultant suspension was filtered, washed by double distilled water, and dried at 105 °C for 12 h. The dried product then was calcined in the muffle furnace at 450 °C for 5 h in (3 °C/min) order to recrystallise the TiO_2_, followed by natural cooling. After calcination, the residue was stored in an airtight container. Pure TiO_2_ was prepared by the same procedure but without the addition of calcined vetiver.

### 2.3. Characterisation

For both pure TiO_2_ and the TiO_2_-vetiver core–shell, the following characterisation was undertaken. The mineralogies were determined by X-ray diffraction (XRD, Philips X’ Pert PRO PW 3719). The crystallite size of particle was calculated using the Scherrer’s equation [37]. The band gaps were calculated from data obtained by UV-Vis spectrophotometry from the diffuse reflectance spectra (DRS, Shimadzu UV-3600) with an integrating sphere attachment (Shimadzu ISR-3100) and the application of the Kubelka–Munk equation [38]. Photoluminescence spectra (PL) were recorded at 300 nm at room temperature a using spectrophotometer (Horiba Jobin Yvon Fluoromax-4). The morphologies were assessed by transmission electron microscopy (TEM, JEOL JSM-2010). The specific surface areas and pore size distributions were determined by N_2_ gas adsorption-desorption using the Brunauer–Emmett–Teller (BET) method (Quantachrome Adtosorb 1 MP).

### 2.4. Photocatalytic Performance Testing

Methylene blue (MB, Sigma-Aldrich (Singpore), ≥95%) was used as the organic dye pollutant. A typical batch test involved mixing 0.01 g of the synthesised core–shell or pure TiO_2_ with 50 mL of MB aqueous solution of 10^−5^ M concentration and magnetic stirring for 12 h in dark conditions (without UV-A irradiation) in order to adsorb the MB maximally on the particle surfaces. The suspensions then were irradiated by UV-A (two 20 W black lights, 370 nm) for 4 h. The irradiation was from above and the distance to the suspension surface was 15 cm. At periodic intervals of irradiation, a small sample of liquid was removed using a vacuum filter. The removed aliquot then was characterised by UV-Vis spectrophotometry using the standard absorbance intensity of λ_max_ = 664 nm using the UV-visible spectrophotometer.

## 3. Results and Discussion

**Figure 1:**
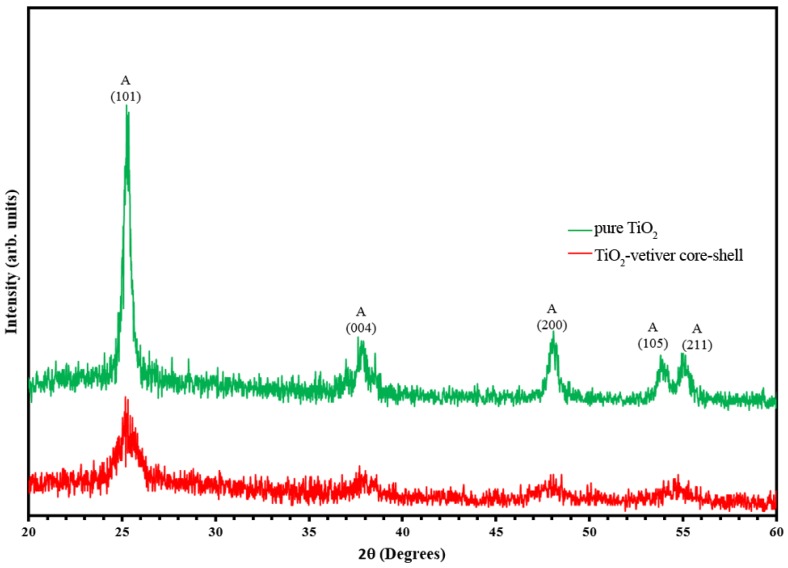
XRD patterns of pure TiO_2_ and TiO_2_-vetiver core–shell.

Figure 1 shows the XRD patterns for pure TiO_2_ and the TiO_2_-vetiver core–shell. The sharp peaks at 25.2°, 37.6°, 48.0°, 54.9°, 62.6°, 70.3°, and 75.0° 2θ for pure TiO_2_ show that it consists solely of the anatase polymorph [39]. In contrast, the TiO_2_-vetiver core–shell peaks also indicate anatase but of lower intensities, which are attributed to the effect of the amorphous residue of the vetiver grass leaves. The reduced intensities, which derived from reduced crystallinity of the anatase, probably resulted from the one or more of the following causes. First, silicon is known as a grain growth inhibitor for TiO_2_ [40], which may have had a similar effect in suppressing nucleation and hence growth. Second, the crystal radii of K and Si in sixfold coordination (0.152 nm and 0.054 nm, respectively) are considerably different from that of Ti (0.0745 nm) [41] and the interstitial site (0.0782 nm) [42]. Consequently, if these dissolved in the TiO_2_ lattice, they would have served to destabilise it. Third, carbon is a very strong reducing agent, so the Ti^4+^ (0.0745 nm) could have been reduced partially to Ti^3+^ (0.081 nm) [41], which could have expanded the lattice at these sites and contracted the lattice at the sites of the charge-compensating oxygen vacancies, which also would have had the potential to destabilise the lattice.

**Figure 2:**
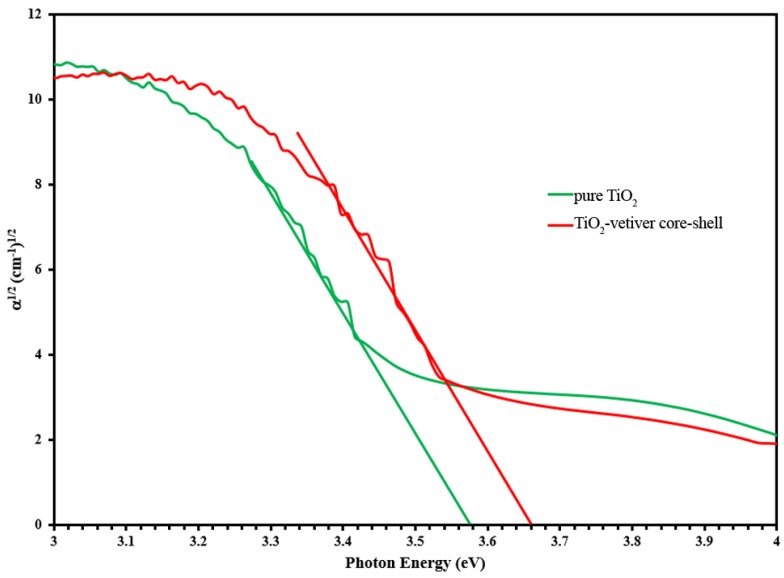
Optical indirect band gaps calculated from DRS data for pure TiO_2_ and TiO_2_-vetiver core–shell.

The data used to calculate the optical indirect band gaps, which are shown in Figure 2, indicate that these are 3.58 eV and 3.66 eV for pure TiO_2_ and the TiO_2_-vetiver core–shell, respectively. The band gap of the former was higher than the commonly reported value of 3.2 eV [9]. However, the band gap of anatase TiO_2_ has been reported to cover a wide range of 3.23–3.59 eV [39]. Although the determined value is within this range, it is possible that it reflects silicon contamination, which is known to increase the band gap [9,43]. The even higher band gap of the TiO_2_-vetiver core–shell is likely to derive from the previously mentioned effect of carbon in the partial reduction of TiO_2_. Consequently, the XRD and band gap data are mutually supportive in suggesting that the presence of carbon caused lattice destabilisation and partial amorphisation. This is supported by modeling of the band gaps of both reduced TiO_2_, which contains oxygen vacancies [44] and of amorphous TiO_2_ [45], which were substantially higher than that of the stoichiometric crystalline analogue.

**Figure 3:**
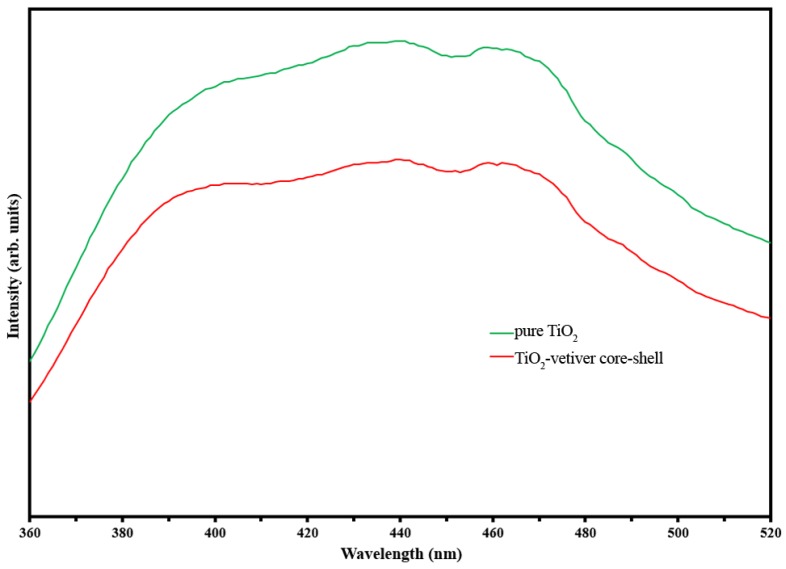
Photoluminescence (PL) spectra of pure TiO_2_ and TiO_2_-vetiver core–shell.

Figure 3 shows photoluminescence (PL) spectra of pure TiO_2_ and the TiO_2_-vetiver core–shell. Since PL emission occurs upon the recombination of the photogenerated electrons and holes [46], then the higher PL intensity of pure TiO_2_ and that of the TiO_2_-vetiver core–shell suggests that the latter experienced enhanced charge separation and so exhibited a reduced recombination rate [47,48]. These effects would increase the probability for electrons and holes to form ROS and hence increase the photocatalytic activity [36,48].

For the TiO_2_-vetiver core–shell, it is likely that reduction by carbon and the consequent generation of oxygen vacancies are responsible for this since it has been reported that oxygen vacancies can enhance charge separation by acting as electron donors [49,50]. It is unlikely that dissolved K or Si played a role since (1) it is highly unlikely that K would dissolve either substitutionally or interstitially owing to size considerations; (2) Si dissolved substitutionally would not affect ionic charge compensation and hence create no oxygen vacancies; and (3) Si dissolved interstitially is likely to have resulted in ionic charge compensation by the formation of Ti vacancies. While Ti vacancies could have acted as hole donors, it is considered that the displacement of Ti^4+^ by Si^4+^ and the resultant formation of Ti vacancies are energetically unfavorable. It has to be noted that, in this work, the increasing of oxygen vacancy has not been directly observed. The increase of oxygen vacancy is implied from the increasing of surface area, which can lead to increasing the surface defects and oxygen vacancy [51,52,53].

**Figure 4:**
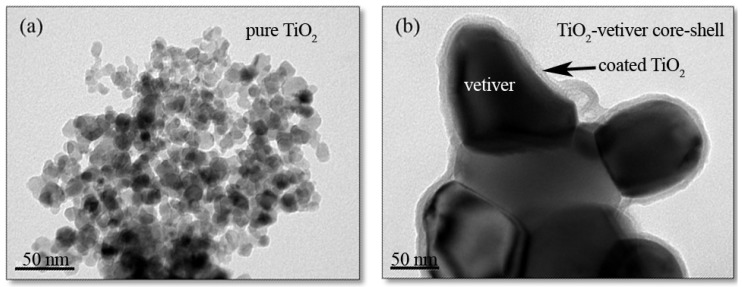
TEM images of (**a**) pure TiO_2_; and (**b**) TiO_2_-vetiver core–shell.

Figure 4 shows TEM images of pure TiO_2_ and the TiO_2_-vetiver core–shell. The pure TiO_2_ was equiaxed and of particle size in the range 10–20 nm, albeit highly agglomerated. It has to be noted that the particle size is very close to the crystallite size, as shown in Table 1. It can be said that the pure TiO_2_ is single grain particle. More interestingly, the TiO_2_-vetiver core–shell exhibited a core–shell structure, where the vetiver acted as the core and the TiO_2_ acted as the shell. The size of the TiO_2_-vetiver core–shell was variable, depending on the nature of the vetiver substrate. The TiO_2_ shell was continuous and of thickness in the range 10–15 nm.

Such core–shell structures have gained considerable interest owing to their ability to enhance both charge separation and resistance to photocorrosion. Analogues of the present core–shell structure exist in the form of TiO_2_ on the surfaces of carbon nanotubes (CNT) [54], TiO_2_ coated on amorphous carbon [55], and the converse arrangement of a TiO_2_ core and graphitic carbon shell [56].

**Figure 5:**
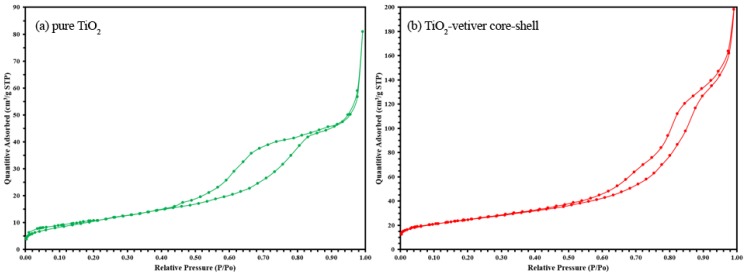
Nitrogen adsorption–desorption isotherm plots for (**a**) pure TiO_2_; and (**b**) TiO_2_-vetiver core–shell.

N_2_ adsorption–desorption isotherms of the pure TiO_2_ and TiO_2_-vetiver core–shell were also studied, as shown in Figure 5. The gas-adsorption isotherm is reported as the volume of gas adsorbed as a function of P/Po. The results suggest that the prepared samples own typical Type IV isotherm. The gap between equilibrium adsorption and desorption pressures above P/Po of about 0.4, further confirming the characteristic of the mesoporous material.

The results for the specific surface area and pore volume of pure TiO_2_ and the TiO_2_-vetiver core–shell are given in Table 1. It can be seen that the values for the pure TiO_2_ are less than half those of the TiO_2_-vetiver core–shell. The evidence for the reason for these differences lies in the nature of the TiO_2_ shell of the TiO_2_-vetiver core–shell. While the pure TiO_2_ appears to have a considerably greater specific surface area, even with the agglomeration, as well as greater pore volume, the TiO_2_ shell of the TiO_2_-vetiver core–shell appears to be smooth. This apparent contradiction can be explained by closer examination of the TiO_2_ shell, which reveals a granular nanostructure that exhibits both surface topography and a network of spherical pores in the sub-nanometre size range. The unexpected presence of the pores is likely to have resulted from the oxidation of the carbon during annealing, which would have disturbed the establishment of the solid nanostructure upon recrystallisation of the TiO_2_. Since the pores appear to be spherical, this suggests gas entrapment during the process. It also is possible that the pores were ruptured and hence continuous, which would increase the specific surface area further.

**Figure 6:**
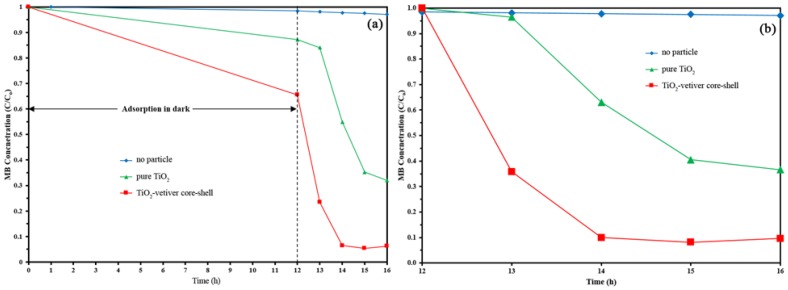
Photocatalytic activity of pure TiO_2_ and TiO_2_-vetiver core–shells, showing (**a**) data for complete test sequence; and (**b**) data normalised following MB adsorption.

Figure 6a shows the complete data for MB adsorption followed by MB degradation. These data confirm that the pure TiO_2_ has less than half the surface area of the TiO_2_-vetiver core–shell. Figure 6b shows the normalised data for MB degradation, where it can be seen that the extent of MB degradation by the TiO_2_-vetiver core–shell effectively was complete within 2 h. The pure TiO_2_ appears to have experienced a 1 h time lag, after which the kinetics of MB degradation were similar to those of the TiO_2_-vetiver core–shell, although the process does not appear to have reached steady-state after 4 h. It is clear that both photocatalyst types perform considerably better under UV-A light.

**Figure 7:**
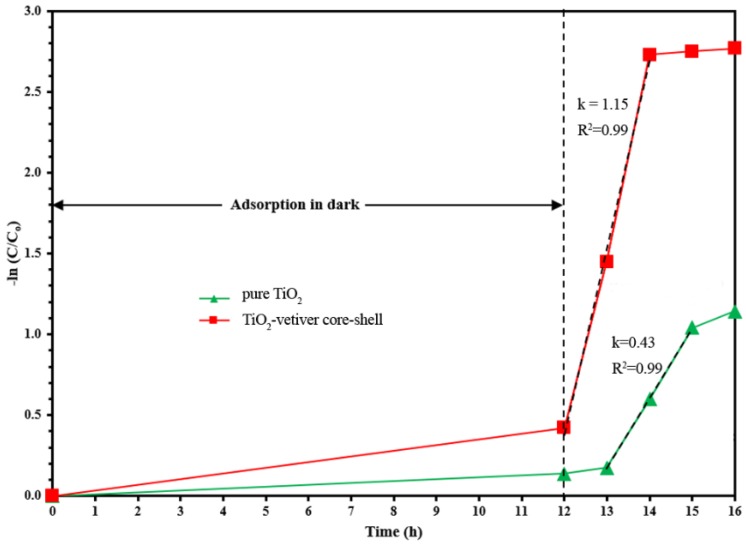
The kinetic of methylene blue degradation.

The preceding data suggest that the photocatalytic activity of the pure TiO_2_ is somewhat inferior to that of the TiO_2_-vetiver core–shell. This observation is consistent with the findings for the photoluminence (Figure 3) and specific surface area data (Table 1), but it contradicts the data for the X-ray diffraction (Figure 1) and the band gap (Figure 2). From this, it is concluded that the controlling factors in the performance depend more on a reduced recombination rate (photoluminescence) and the number of surface active sites (specific surface area) rather than the crystallinity (X-ray diffraction) or the semiconducting and optical properties (band gap). In addition, the charge carrier diffusion distance in the TiO_2_-vetiver core–shell would be relatively short owing to the 10–15 nm shell thickness and the possibility that it could be even less owing to the thinner walls resulting from the porosity. The diffusion distance in the pure TiO_2_ would be longer owing to the larger 10–20 nm (solid) grain size. It is well known that the recombination rate decreases with decreasing charge carrier diffusion distance. Figure 7 shows the kinetic rate of MB removal. It can be seen that after irradiation the MB was completely remove in 2 h by TiO_2_-vetiver core–shell. Since the TiO_2_-vetiver core–shell has higher surface area and volume of pores, then this can result higher adsorption (Figure 6a). Then, during the photocatalytic mechanism (after irradiation), the MB at the surface could be decomposed quickly so the surface can adsorb more MB. It can be said that the co-mechanisms of adsorption by adsorbent and photocatalysis lead to higher performance compared to only photocatalytic of pure TiO_2_.

**Figure 8:**
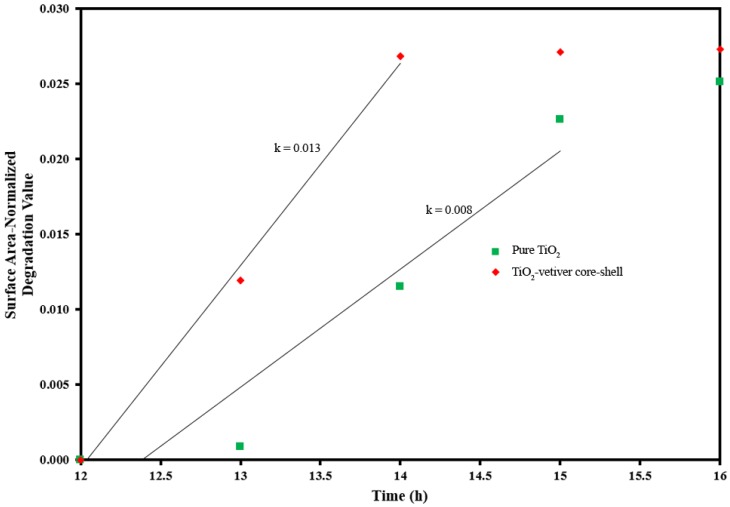
Kinetics plots of the surface area-normalised degradation values against light irradiation time.

Data provides the surface area-normalised degradation constants. The comparison of normalised degradation constants excluded the influence of surface areas. In order to investigate the effect of surface area from the adsorbent on the degradation activity of TiO_2_, the surface area-normalised degradation values against light irradiation time were plotted as shown in Figure 8 and the calculated surface area-normalised rate constants are presented in Table 1. The results clearly suggested that surface area of the catalyst has a crucial impact on the activity of degradation in this study because the surface area-normalised rate constants of TiO_2_-vetiver core–shell are increased from the pure TiO_2_.

**Figure 9:**
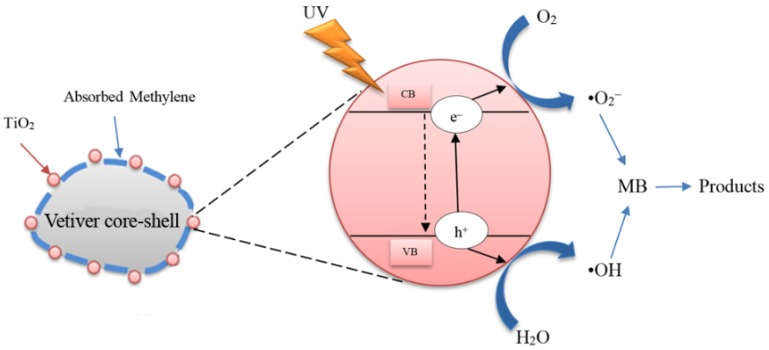
The schematic representation of the photocatalytic mechanism.

The schematic representation of the photocatalytic mechanism is present in Figure 9. It can be explained that during the adsorption process MB can be adsorbed at the surface of TiO_2_-vetiver core–shell as can be seen in Figure 5a. After irradiation, the photocatalytic at the surface of TiO_2_-vetiver core–shell can occur immediately. This results in enhancing the photocatalytic performance of MB degradation.

## 4. Conclusions

The present work reports a comparison between nanoparticles of pure TiO_2_ and a core–shell structure of TiO_2_ on calcined vetiver grass leaves. The samples were fabricated using a sol-gel method that involved heating at 450 °C for 5 h, which yielded the anatase polymorph of TiO_2_. The comparison was based on data for X-ray diffraction, UV-Vis spectrophotometry, photoluminescence, transmission electron microscopy, specific surface area measurement, pore volume assessment, and methylene blue degradation testing.

The results showed that the pure TiO_2_ consisted of agglomerated equiaxed nanoparticles of individual grain sizes in the range 10–20 nm. In contrast, the TiO_2_-vetiver composite exhibited a core–shell structure consisting of a carbonaceous core and TiO_2_ shell of thickness 10–15 nm. The TiO_2_ shell formed a continuous coating with topography and porous network at a sub-nanometre scale. These features appear to have had a significant influence on the photocatalytic performance in that, compared to the pure TiO_2_, the lower cross-sectional area, greater surface area, and higher pore volume of the TiO_2_ shell potentially increased the number of active sites, reduced the charge carrier diffusion distance, and reduced the recombination rate, thereby improving the photocatalytic activity. This improvement derived from morphological characteristics rather than crystallographic, semiconducting, or optical properties.

The improved performance of the TiO_2_-vetiver core–shell was unexpected in light of the X-ray diffraction data, which showed that the crystallinity of the TiO_2_ was lower than that of the pure TiO_2_. It also was unexpected in consideration of the unusually high band gap of the TiO_2_ shell. However, both of these outcomes are likely to have been generated through the reducing effect of the carbon on the TiO_2_ during heating, thereby facilitating the formation of oxygen vacancies, which enhance charge separation and hence photocatalysis by TiO_2_. In this sense, the effect of charge separation dominated those of crystallinity and band gap. It has to be noted that, in this work, the increasing of oxygen vacancy has not been directly observed. The increase of oxygen vacancy is implied from the increasing of surface area, which can lead to increasing the surface defects and oxygen vacancy.

## Figures and Tables

**Table 1:** BET data for surface properties of pure TiO_2_ and TiO_2_-vetiver core–shell.

Sample	Specific Surface Area (m^2^/g)	Pore Volume (cm^3^/g)	Crystallite Size at (101) nm	Surface Area-Normalised Rate Constants (h^−1^·m^−2^·g)
Pure TiO_2_	40	0.1252	21	0.008
TiO_2_-Vetiver Core–shell	86	0.3065	14	0.013

## References

[1] (2012). Managing Water Uncertainty and Risk.

[2] Vo P.T., Ngo H.H., Guo W., Zhou J.L., Nguyen P.D., Listowski A., Wang X.C. (2014). A mini-review on the impacts of climate change on wastewater reclamation and reuse. Sci. Total Environ..

[3] Visa M., Andronic L., Duta A. (2015). Fly ash-TiO_2_ nanocomposite material for multi-pollutants wastewater treatment. J. Environ. Manag..

[4] Asghar A., Raman A.A.A., Daud W.M.A.W. (2015). Advanced oxidation processes for in-situ production of hydrogen peroxide/hydroxyl radical for textile wastewater treatment: A review. J. Clean Prod..

[5] Pang Y.L., Abdullah A.Z. (2013). Fe^3+^ doped TiO_2_ nanotubes for combined adsorption–sonocatalytic degradation of real textile wastewater. Appl. Catal. B.

[6] United States Environmental Protection Agency (EPA) (2012). Guidelines for Water Reuse.

[7] De la Cruz N., Romero V., Dantas R.F., Marco P., Bayarri B., Giménez J., Esplugas S. (2013). o-Nitrobenzaldehyde actinometry in the presence of suspended TiO_2_ for photocatalytic reactors. Catal. Today.

[8] Zhang Z., Wu H., Yuan Y., Fang Y., Jin L. (2012). Development of a novel capillary array photocatalytic reactor and application for degradation of azo dye. Chem. Eng. J..

[9] Nakaruk A., Ragazzon D., Sorrell C.C. (2010). Anatase–rutile transformation through high-temperature annealing of titania films produced by ultrasonic spray pyrolysis. Thin Solid Films.

[10] Mills A., O’Rourke C., Moore K. (2015). Powder semiconductor photocatalysis in aqueous solution: An overview of kinetics-based reaction mechanisms. J. Photochem. Photobiol. A.

[11] Pelaez M., Nolan N.T., Pillai S.C., Seery M.K., Falaras P., Kontos A.G., Dunlop P.S.M., Hamilton J.W.J., Byrnee J.A., O’Shea K. (2012). A review on the visible light active titanium dioxide photocatalysts for environmental applications. Appl. Catal. B.

[12] Zangeneh H., Zinatizadeh A.A.L., Habibi M., Akia M., Hasnain Isa M. (2015). Photocatalytic oxidation of organic dyes and pollutants in wastewater using different modified titanium dioxides: A comparative review. J. Ind. Eng. Chem..

[13] Spasiano D., Marotta R., Malato S., Fernandez-Ibanez P., Somma I.D. (2015). Solar photocatalysis: Materials, reactors, some commercial, and pre-industrialized applications. A comprehensive approach. Appl. Catal. B.

[14] Cardoso J.C., Lucchiari N., Zanoni M.V.B. (2015). Bubble annular photoeletrocatalytic reactor with TiO_2_ nanotubes arrays applied in the textile wastewater. J. Environ. Chem. Eng..

[15] Kılıç B., Gedik N., Pıravadıllı Mucur S., Serhan Hergula A., Gür E. (2015). Band gap engineering and modifying surface of TiO_2_ nanostructures by Fe_2_O_3_ for enhanced-performance of dye sensitized solar cell. Mater. Sci. Semicon. Proc..

[16] Rico V., Romero P., Hueso J.L., Espinós J.P., González-Elipe A.R. (2009). Wetting angles and photocatalytic activities of illuminated TiO_2_ thin films. Catal. Today.

[17] Dittricha T., Ofir A., Tirosh S., Grinis L., Zaban A. (2006). Influence of the porosity on diffusion and lifetime in porous TiO_2_ layers. Appl. Phys. Lett..

[18] Romero-Gómez P., Hamad S., González J.C., Barranco A., Espinós J.P., Cotrino J., González-Elipe A.R. (2010). Band gap narrowing versus formation of electronic states in the gap in N−TiO_2_ thin films. J. Phys. Chem. C.

[19] Romero-Gómez P., Rico V., Borrás A., Barranco A., Espinós J.P., Cotrino J., González-Elipe A.R. (2009). Chemical state of nitrogen and visible surface and Schottky barrier driven photoactivities of N-doped TiO_2_ thin films. J. Phys. Chem. C.

[20] Bulushev D.A., Kiwi-Minsker L., Zaikovskii V.I., Lapina O.B., Ivanov A.A., Reshetnikov S.I., Renken A. (2000). Effect of potassium doping on the structural and catalytic properties of V/Ti-oxide in selective toluene oxidation. Appl. Catal. A.

[21] Phanichphant S., Nakaruk A., Channei D. (2016). Photocatalytic activity of the binary composite CeO_2_/SiO_2_ for degradation of dye. Appl. Surf. Sci..

[22] Chong M.N., Jin B., Chow C.W.K., Saint C. (2010). Recent developments in photocatalytic water treatment technology: A review. Water Res..

[23] Liu S., Lim M. (2014). Amal, R. TiO_2_-coated natural zeolite: Rapid humic acid adsorption and effective photocatalytic regeneration. Chem. Eng. Sci..

[24] Moustakas N.G., Kontos A.G., Likodimos V., Katsaros F., Boukos N., Tsoutsou D., Dimoulas A., Romanos G.E., Dionysiou D.D., Falaras P. (2013). Inorganic–organic core–shell titania nanoparticles for efficient visible light activated photocatalysis. Appl. Catal. B.

[25] Su T., Chen S., Quan X., Zhao H., Zhang Y. (2008). A silicon-doped TiO_2_ nanotube arrays electrode with enhanced photoelectrocatalytic activity. Appl. Surf. Sci..

[26] Courcot D., Grzybowska B., Barbaux Y., Rigole M., Ponchel A., Guelton M. (1996). Effect of potassium addition to the TiO_2_ support on the structure of V_2_O_5_/TiO_2_ and its catalytic properties in the oxidative dehydrogenation of propane. J. Chem. Soc. Faraday Trans..

[27] Kanakaraju D., Kockler J., Motti C.A., Glass B.D., Oelgemoller M. (2015). Titanium dioxide/zeolite integrated photocatalytic adsorbents for the degradation of amoxicillin. Appl. Catal. B.

[28] Ghorbani F., Younesi H., Mehraban Z., Celik M.S., Ghoreyshi A.A., Anbia M. (2013). Preparation and characterization of highly pure silica from sedge as agricultural waste and its utilization in the synthesis of mesoporous silica MCM-41. J. Taiwan Inst. Chem. Eng..

[29] Chen Y.D., Huang M.J., Huang B., Chen X.R. (2012). Mesoporous activated carbon from inherently potassium-rich pokeweed by in situ self-activation and its use for phenol removal. J. Anal. Appl. Pyrolysis.

[30] Ye M., Sun M., Liu Z., Ni N., Chen Y., Gu C., Kengara F.O., Li H., Jiang X. (2014). Evaluation of enhanced soil washing process and phytoremediation with maize oil, carboxymethyl-b-cyclodextrin, and vetiver grass for the recovery of organochlorine pesticides and heavy metals from a pesticide factory site. J. Environ. Manage..

[31] Paillat L., Périchet C., Pierrat J., Lavoine S., Filippi J., Meierhenrich U., Fernandez X. (2012). Purification of vetiver alcohols and esters for quantitative high-performance thin-layer chromatography determination in Haitian vetiver essential oils and vetiver acetates. J. Chromatogr. A.

[32] Yaseen M., Singh M., Ram D. (2014). Growth, yield and economics of vetiver (*Vetiveria zizanioides* L. Nash) under intercropping system. Ind. Crops Prod..

[33] Singh R., Narzary D., Bhardwaj J., Singh A.K., Kumar S., Kumar A. (2014). Molecular diversity and SSR transferability studies in Vetiver grass (*Vetiveria zizanioides* L. Nash). Ind. Crops Prod..

[34] Lal R.K., Gupta P., Gupta V., Sarkar S., Singh S. (2013). Genetic variability and character associations in vetiver (*Vetiveria zizanioides* L. Nash). Ind. Crops Prod..

[35] Le S.T.T., Yuangpho N., Threrujirapapong T., Khanitchaidecha W., Nakaruk A. (2015). Synthesis of mesoporous materials from vetiver grass for wastewater treatment. J. Aust. Ceram. Soc..

[36] Rajamanickam D., Shanthi M. (2014). Photocatalytic degradation of an azo dye Sunset Yellow under UV-A light using TiO_2_/CAC composite catalysts. Spectrochim. Acta, Part A.

[37] Dai S., Wu Y., Sakai T., Du Z., Sakai H., Abe M. (2010). Preparation of highly crystalline TiO_2_ nanostructures by acid-assisted hydrothermal treatment of hexagonal-structured nanocrystalline titania/cetyltrimethyammonium bromide nanoskeleton. Nanoscale Res. Lett..

[38] Bezares I., del Campo A., Herrasti P., Muñoz-Bonilla A. (2015). A simple aqueous electrochemical method to synthesize TiO_2_ nanoparticles. Phys. Chem. Chem. Phys..

[39] Hanaor D.A.H., Sorrell C.C. (2011). Review of the anatase to rutile phase transformation. J. Mater. Sci..

[40] Lin C.P., Chen H., Nakaruk A., Koshy P., Sorrell C.C. (2013). Effect of annealing temperature on the photocatalytic activity of TiO_2_ thin films. Energy Procedia.

[41] Shannon R.D. (1976). Revised effective ionic radii and systematic studies of interatomic distances in halides and chalcogenides. Acta Crystallogr. A.

[42] Chen W.F., Koshy P., Huang Y., Adabifiroozjaei E., Yao Y., Sorrell C.C. (2016). Effects of precipitation, liquid formation, and intervalence charge transfer on the properties and photocatalytic performance of cobalt- or vanadium-doped TiO_2_ thin films. Int. J. Hydrogen Energy.

[43] Nakaruk A., Lin C.Y.W., Koshy P., Sorrell C.C. (2012). Iron doped titania thin films prepared by spin coating. Adv. Appl. Ceram..

[44] Lin Z., Orlov A., Lambert R.M., Payne M.C. (2005). New insights into the origin of visible light photocatalytic activity of nitrogen-doped and oxygen-deficient anatase TiO_2_. J. Phys. Chem. B.

[45] Prasai B., Cai B., Underwood M.K., Lewis J.P., Drabold D.A. (2012). Properties of amorphous and crystalline titanium dioxide from first principles. J. Mater. Sci..

[46] Yu J.G., Yu H.G., Cheng B., Zhao X.J., Yu J.C., Ho W.K. (2003). The effect of calcination temperature on the surface microstructure and photocatalytic activity of TiO_2_ thin films prepared by liquid phase deposition. J. Phys. Chem. B.

[47] Zhang F., Maeda K., Takata T., Domen K. (2011). Improvement of the photocatalytic hydrogen evolution activity of Sm_2_Ti_2_S_2_O_5_ under visible light by metal ion additives. J. Catal..

[48] Tian F., Wu Z., Chen Q., Yan Y., Cravotto G., Wu Z. (2015). Microwave-induced crystallization of AC/TiO_2_ for improving the performance of rhodamine B dye degradation. Appl. Surf. Sci..

[49] Pesci F.M., Wang G., Klug D.R., Li Y., Cowan A.J. (2013). Efficient suppression of electron–hole recombination in oxygen-deficient hydrogen-treated TiO_2_ nanowires for photoelectrochemical water splitting. J. Phys. Chem. C.

[50] Wang G., Wang H., Ling Y., Tang Y., Yang X., Fitzmorris R.C., Wang C., Zhang J.Z., Li Y. (2011). Hydrogen-treated TiO_2_ nanowire arrays for photoelectrochemical water splitting. Nano Lett..

[51] Zhang J., Zhao Z., Wang X., Yu T., Guan J., Yu Z., Li Z., Zou Z. (2010). Increasing the oxygen vacancy density on the TiO_2_ surface by La-doping for dye-sensitized solar cells. J. Phys. Chem. C.

[52] Fujii K., Sato Y., Takase S., Shimizu Y. (2015). Effects of oxygen vacancies and reaction conditions on oxygen reduction reaction on Pyrochlore-Type lead-ruthenium oxide. J. Electrochem. Soc..

[53] Xu L., Jiang Q., Xiao Z., Li X., Huo J., Wang S., Dai L. (2016). Plasma-engraved Co_3_O_4_ nanosheets with oxygen vacancies and high surface area for the oxygen evolution reaction. Angew. Chem. Int. Ed..

[54] Huang S.H., Wang C.C., Liao S.Y., Gan J.Y., Perng T.P. (2016). CNT/TiO_2_ core–shell structures prepared by atomic layer deposition and characterization of their photocatalytic properties. Thin Solid Films.

[55] Zhang G., Teng F., Zhao C., Chen L., Zhang P., Wang Y., Gong C., Zhang Z., Xie E. (2014). Enhanced photocatalytic activity of TiO_2_/carbon@TiO_2_ core–shell nanocomposite prepared by two-step hydrothermal method. Appl. Surf. Sci..

[56] Lee J.M., Han S.B., Kim J.Y., Lee Y.W., Ko A.R., Roh B., Hwang I., Park K.W. (2010). TiO_2_@carbon core–shell nanostructure supports for platinum and their use for methanol electrooxidation. Carbon.

